# P007: Risk factors for mortality among non-icu patients with catheter-related bacteraemia

**DOI:** 10.1186/2047-2994-2-S1-P7

**Published:** 2013-06-20

**Authors:** A Hornero, E Shaw, R Escofet, T Vidal, C Ardanuy, D Garcia, C Peña, J Ariza, M Pujol

**Affiliations:** 1 Infection Control, Hospital Universitari de Bellvitge, Hospitalet Llobregat, Spain; 2 Microbiology, Hospital Universitari de Bellvitge, Hospitalet Llobregat, Spain

## Introduction

The number of episodes of vascular catheter related bacteraemia (CRB) observed in non-Intensive Care Unit (ICU) patients may be similar or even higher than those observed in ICUs. While we have a lot of information regarding the impact of CRB among ICU patients, there is still lack of information concerning non-ICU patients.

## Objectives

To determine predictors of mortality among non-ICU patients with CRB.

## Methods

From Jan 2003 to Dec 2012, a prospective continuous surveillance of CRB including all adult patients admitted to non-ICU wards for more than 48h, was carried out in a tertiary centre. Monitoring of CRB was performed by daily meeting of Infection Control Team and microbiology department. Patients were visited and those cases that fulfilled criteria for CRB were selected. Patients were followed up until discharge. Mortality was defined as in-hospital death from any cause occurring in the 30 days after the onset of CRB. A logistic regression model was performed to identify risk factors for mortality.

## Results

From 2003 to 2012, 590 episodes of CRB were detected in 578 non-ICU patients; 285 in medical wards and 305 in surgical wards. Mean age was 64y (SD 14y) and 37% were females. Mortality was 16.1%. Among all episodes of CRB, 332 (56%) were caused by central venous catheter (19% subclavian, 17% jugular, 12% femoral, 8% peripheral inserted central catheter) and 258 (44%) by peripheral venous catheter. Gram positive cocii caused 72% of episodes, gram negative bacilli 28% and fungi 1%. Among them, *S.aureus* was identified in 235 episodes (40%), coagulase negative in 174 (29%), enterococci in 23 (4%), *P.aeruginosa* in 33 (6%) and Candida spp in 7 episodes (1%). Independent risk factors associated to mortality in multivariate analysis were: age older than 65y (OR: 2.0;95% CI:1.2-3.2), hospitalization in medical wards (OR:1.6;95CI:1.0-2.6) and *S.aureus* (OR:3.1;95%CI:1.9-5.0), while type of catheter and place of insertion were not associated.

## Conclusion

Among non-critically-ill patients with CRB, those older than 65y, hospitalized in medical wards and with *S.aureus* aetiology had a greater risk of mortality.

## Disclosure of interest

None declared

